# Lossless quantum data compression with exponential penalization: an operational interpretation of the quantum Rényi entropy

**DOI:** 10.1038/s41598-017-13350-y

**Published:** 2017-11-07

**Authors:** Guido Bellomo, Gustavo M. Bosyk, Federico Holik, Steeve Zozor

**Affiliations:** 1 CONICET-Universidad de Buenos Aires, Instituto de Investigación en Ciencias de la Computación (ICC), Buenos Aires, Argentina; 2 Instituto de Física La Plata, UNLP, CONICET, Facultad de Ciencias Exactas, Casilla de Correo 67, 1900 La Plata La Plata, Argentina; 3 Univ. Grenoble Alpes, CNRS, Grenoble INP Institute of Engineering, GIPSA-Lab, 38000 Grenoble, France

## Abstract

Based on the problem of quantum data compression in a lossless way, we present here an operational interpretation for the family of quantum Rényi entropies. In order to do this, we appeal to a very general quantum encoding scheme that satisfies a quantum version of the Kraft-McMillan inequality. Then, in the standard situation, where one is intended to minimize the usual average length of the quantum codewords, we recover the known results, namely that the von Neumann entropy of the source bounds the average length of the optimal codes. Otherwise, we show that by invoking an exponential average length, related to an exponential penalization over large codewords, the quantum Rényi entropies arise as the natural quantities relating the optimal encoding schemes with the source description, playing an analogous role to that of von Neumann entropy.

## Introduction

One of the main concerns in classical and quantum information theory is the problem of encoding information by using fewest resources as possible. This task is known as *data compression* and it can be carried out either in a *lossy* or a *lossless* way, depending on whether the original data can be recovered with or without errors, respectively.

Here, we are interested in lossless *quantum* data compression. In order to state our proposal, let us first recall how this task works in the classical domain. The mathematical foundations of classical data compression can be found in the seminal paper of Shannon^1^ (see e.g.^2^ for an introduction to the topic), although we can summarize it as follows. Let $S=\{{p}_{i},{s}_{i}\}$ be a classical source where each symbol *s*_*i*_ has associated a probability of occurrence *p*_*i*_. The idea is to assign to each symbol a codeword $c({s}_{i})$ of some alphabet $A=\mathrm{\{0,}\ldots ,k-\mathrm{1\}}$ in an adequate way. In particular, a *k*-ary classical code *c* of *S* is said *uniquely decodable* if this assignment of codewords is injective for any possible concatenation. A celebrated result states that any uniquely decodable code necessarily satisfies the *Kraft-McMillan inequality* ^3,4^: ${\sum }_{i}{k}^{-{\ell }_{i}}\le 1$ where ${\ell }_{i}$ is the length of the codeword $c({s}_{i})$ (measured in bits if *k* = 2). Conversely, given a set of codewords lengths $\{{\ell }_{i}\}$, there exists a uniquely decodable code with these lengths. Thus, lossless data compression consists in finding a uniquely decodable code taking into account the statistical description of the source. Formally, this is carried out by minimizing the average codeword length $L={\sum }_{i}{p}_{i}{\ell }_{i}$ subject to the Kraft-McMillan inequality. In the end, one obtains a *variable-length* code where shorter codewords are assigned to symbols with a high probability of occurrence, whereas larger codewords are assigned to symbols with low probability (see^2^, chap. 5). Moreover, one has that (in the limit of the large number of independent and identically-distributed sources) the average length of the optimal code is arbitrarily close to the *Shannon entropy* ^1^ of the source, $H(p)=-{\sum }_{i}{p}_{i}{\mathrm{log}}_{k}{p}_{i}$.

As noticed by Campbell, the previous solution has the disadvantage that it can happen that the codeword length turns out to be very large for symbols with a sufficiently low probability of occurrence^5^. Indeed, the use of average codewords length as a criterion of performance has the implicit assumption that the cost varies linearly with the codeword length, which is not always desirable. For instance, it could be the case that adding a letter to a large codeword may have a larger impact than adding a letter to a shorter codeword, for instance in terms of memory needed to store a codeword. This problem has given place to the proposal of several other measures of codeword lengths (see e.g.^6–9^), for which the average length is a limiting case. In particular, a generalized average *t*-length, also called exponential average, is defined as^5^ ${L}_{t}=\frac{1}{t}{\sum }_{i}{\mathrm{log}}_{k}{\sum }_{i}{p}_{i}{k}^{t{\ell }_{i}}$, where $t\ge 0$ is a parameter related to the cost. Notice that in the limiting case $t\to 0$ one recovers ${L}_{t}\to L$ and, as *t* increases, a greater *penalization* over the large codewords holds. Indeed, Campbell has obtained a source coding theorem taking into account such a penalization. His theorem is similar to the standard one, but the encoding is made in such a way that the generalized codeword length turns out to be arbitrarily close to the *Rényi entropy* ^10^ of the source, ${H}_{\alpha }(p)=\frac{1}{1-\alpha }{\mathrm{log}}_{k}{\sum }_{i}{p}_{i}^{\alpha }$ with $\alpha =\frac{1}{t+1}$. This remarkable result provides an operational interpretation of the Rényi entropy as the natural information measure for the problem of optimal data compression with penalization over large codewords (see also^11^ for a discussion of an axiomatic derivation of entropy related to the coding problem).

As we have seen, variable-length codes arise naturally in the problem of lossless classical data compression. In the quantum information theory realm, the formulation of this problem presents intrinsic difficulties. These difficulties are mainly related to the fact that a quantum source can possibly send mutually non-orthogonal states. Thus, one has to deal with superpositions of quantum codewords. Even worse, these superpositions may correspond to codewords of different lengths. Schumacher and Westmoreland were the first in establishing a general approach to the problem of quantum variable-length coding^12^. Furthermore, they have provided the first quantum version of the Kraft-McMillan inequality and have found that the von Neumann entropy of the source *ρ*, $S(\rho )=-\mathrm{Tr}(\rho {\mathrm{log}}_{2}\rho )$ (binary logarithm for coding in qubits), plays an analogous role to that of the Shannon entropy in the classical source coding theorem. Several other authors have contributed to this subject proposing alternative or extended schemes^12–20^. In general, these approaches face the same disadvantage as in the classical case: namely they do not consider the fact that large codewords, even appearing with low probabilities, may have large impact in terms of resources needed for the encoding. This drawback is even more relevant nowadays, due to the fact that the practical implementation of quantum information protocols pose the challenge of manipulating coherent superpositions of qubits. While the use of chains of qubits of arbitrary length may arise naturally in some theoretical considerations, it can be very expensive and difficult to implement large chains in the lab, specially at the early stages of the development of quantum information technology devices. Thus, our goal is to provide a quantum version of Campbell’s strategy for the problem of coding with penalization of large codewords. As a consequence, we show that in this framework the quantum Rényi entropies emerge as the natural quantities relating the optimal encoding schemes with the source description. Accordingly, we provide an operational interpretation for those entropies.

## Results

### Uniquely decodable quantum code and quantum Kraft-McMillan inequality

In this section, we summarize some definitions and results of the literature related to our proposal. We begin by pointing out the problem of *lossless quantum compression*.

Lossless quantum compression consists in compressing a quantum source given by an ensemble of quantum states, by using a variable-length quantum code so that the original states can be exactly recovered, *i.e*., without error. More precisely, the situation to deal with is the following. Let us assume that a quantum source produces an ensemble of quantum states ${\mathscr{S}}={\{{p}_{n},|{s}_{n}\rangle \}}_{n=1}^{N}$, where ${p}_{n}\ge 0$, ${\sum }_{n\mathrm{=1}}^{N}{p}_{n}\mathrm{=1}$ and $|{s}_{n}\rangle \in \,{ {\mathcal H} }_{{\mathscr{S}}}\,\equiv {{\mathbb{C}}}^{d}$. The first task is to encode in an unambiguous or uniquely decodable way not only every single quantum state $|{s}_{n}\rangle$ of the source, but also any string of quantum states of the source. In this sense, let us first introduce a very general definition of a *uniquely decodable quantum source code*.

#### Definition 1.

A uniquely decodable quantum source code of ${\mathscr{S}}$ over a quantum *k*-ary alphabet ${\mathscr{A}}=\{|0\rangle ,$ $\ldots ,|k-1\rangle \}\,\subset \,{ {\mathcal H} }_{{\mathscr{A}}}\,\equiv \,{{\mathbb{C}}}^{k}$, with $k\in {{\mathbb{N}}}^{\ast }\backslash \mathrm{\{1\}}$, is a linear isometry map $U\,:{ {\mathcal F} }_{{\mathscr{S}}}\to { {\mathcal F} }_{{\mathscr{A}}}$ where ${ {\mathcal F} }_{{\mathscr{X}}}\equiv {\oplus }_{\ell \mathrm{=0}}^{\infty }{ {\mathcal H} }_{{\mathscr{X}}}^{\,\otimes \ell }$ is a Fock space, where ${\mathscr{X}}={\mathscr{S}}$ or ${\mathscr{A}}$.

In this way, the fact that *U* is an isometry guarantees an injective mapping which assigns for each string of the form ${\otimes }_{m=1}^{M}|{s}_{{i}_{m}}\rangle$, with ${i}_{m}\in \mathrm{\{1,}\ldots ,N\}$ and $M\in {{\mathbb{N}}}^{\ast }$, a quantum codeword $U{\otimes }_{m\mathrm{=1}}^{M}|{s}_{{i}_{m}}\rangle \in { {\mathcal F} }_{{\mathscr{A}}}$. Let us see how our definition works for single code words. A single quantum codeword over ${\mathscr{A}}$ is a quantum pure state that belongs to the Fock space ${ {\mathcal F} }_{{\mathscr{A}}}$ (we are taking here strings with a single component). Thus, we can write ${\rm{U}}|{{\rm{s}}}_{n}\rangle$ $=\,{\sum }_{j}{a}_{j,n}|{a}_{j,n}\rangle$, where ${a}_{j,n}\in {\mathbb{C}}$, ${\sum }_{j}|{a}_{j,n}{|}^{2}=1$ and $|{a}_{j,n}\rangle \in { {\mathcal H} }_{{\mathscr{A}}}^{\otimes {l}_{j}}\subset { {\mathcal F} }_{{\mathscr{A}}}$. Notice that the number of non-vanishing coefficients in the set ${\{{a}_{j,n}\}}_{j\mathrm{=1}}^{\infty }$ could be infinite in principle. In the following we will restrict to the finite case (*i.e*., ${a}_{j,n}=0$ for almost all *j*).

Up to now, we have given a very formal definition of uniquely decodable quantum source code. In order to show an encoding scheme that satisfies definition 1, we mainly follow the proposal given in^19,20^. First, let us precise the definition of a *k*-ary classical uniquely decodable code for the symbols source $S=\mathrm{\{1},\ldots ,d\}$ over an alphabet $A=\mathrm{\{0},\ldots ,k-\mathrm{1\}}$. Let *F*_*A*_ be the set ${F}_{A}={\cup }_{\ell \mathrm{=0}}^{\infty }{A}^{\ell }$. Then, $c\,:S\to {F}_{A}$ is a classical uniquely decodable code if and only if for any $M\ge 1$, any concatenation ${c}^{M}({i}_{1},\ldots ,{i}_{M})=c({i}_{1})\cdots c({i}_{M})$ of *M* codewords is an injective function (see e.g.^2^). We denote by ${\ell }_{i}$ the length of the *i*-th codeword, *i.e*., the number of “letters” of *A* appearing in the codeword $c(i)$. Hereafter, we consider the isometries $U\,:{ {\mathcal H} }_{{\mathscr{S}}}\to { {\mathcal F} }_{{\mathscr{A}}}$ of the form^19,20^,

1 $$U=\sum _{i\mathrm{=1}}^{d}|c(i)\rangle \langle {e}_{i}|,$$

where ${\{|{e}_{i}\rangle \}}_{i\mathrm{=1}}^{d}$ is a basis of ${ {\mathcal H} }_{{\mathscr{S}}}$ and *c* a classical uniquely decodable code of *S*. Clearly, by construction, one has $|c(i)\rangle$ $\in { {\mathcal H} }_{{\mathscr{A}}}^{\otimes {\ell }_{i}}\,\subset \,{ {\mathcal F} }_{{\mathscr{A}}}$ and $\{|c(i)\rangle \}$ forms an orthonormal set, so that ${U}^{\dagger }U=I$ (but notice that, in general, $U{U}^{\dagger }$ can be different from the identity operator). We refer any isometry of the form (1) as *lossless quantum encoding scheme*. Note that contrary to a classical code, | $|c(i)\rangle$ here does not encode any quantum state of the source $\{|{s}_{n}\rangle \}$ but the base state $|{e}_{i}\rangle$, except when $|{s}_{n}\rangle =|{e}_{i}\rangle$ for some $n,i$. As introduced, the codeword associated to a superposition of source states is the superposition of the codewords. Moreover, $U|{s}_{n}\rangle$ does not necessarily belong to a space of the form ${ {\mathcal H} }_{{\mathscr{A}}}^{\,\otimes \ell }$ for some $\ell$. Notice now that a quantum coding scheme *U* can be extended to a map ${U}^{M}\,:{ {\mathcal H} }_{{\mathscr{S}}}^{\,\otimes M}\to { {\mathcal F} }_{{\mathscr{A}}}$ on sentences a follows:

2 $${U}^{M}=\sum _{{i}_{1}\mathrm{=1}}^{d}\cdots \sum _{{i}_{M}\mathrm{=1}}^{d}|c({i}_{1})\cdots c({i}_{M})\rangle \langle {e}_{({i}_{1})}\cdots {e}_{({i}_{M})}|\mathrm{.}$$

The above map is well defined for all $M\in {{\mathbb{N}}}^{\ast }$. A map such as *U* ^*M*^ can be naturally considered as an operator acting in the Fock space ${ {\mathcal F} }_{{\mathscr{S}}}$ by viewing $|{e}_{{i}_{1}}\cdots {e}_{{i}_{M}}\rangle \in { {\mathcal H} }_{{\mathscr{S}}}^{\,\otimes M}\subset { {\mathcal F} }_{{\mathscr{S}}}$ as follows. Consider a state $|\varphi \rangle \in { {\mathcal H} }_{{\mathscr{S}}}^{\,\otimes M^{\prime} }$. Then, we write ${U}^{M}|\varphi \rangle ={\delta }_{M,{M}^{^{\prime} }}{\sum }_{{i}_{1}\mathrm{=1}}^{d}\cdots {\sum }_{{i}_{M}\mathrm{=1}}^{d}\langle {e}_{{i}_{1}}\cdots {e}_{{i}_{M}}|\varphi \rangle |c({i}_{1})\cdots c({i}_{M})\rangle$. Now, with this observation we can define an operator ${U}^{\infty }\,:{ {\mathcal F} }_{{\mathscr{S}}}\to { {\mathcal F} }_{{\mathscr{A}}}$ as

3 $${U}^{\infty }=\sum _{M\mathrm{=1}}^{\infty }{U}^{M}\mathrm{.}$$

The physical interpretation of ${U}^{\infty }$ is that for each sentence ${\otimes }_{m\mathrm{=1}}^{M}|{s}_{{i}_{m}}\rangle$ of the source, we will obtain the right coded sentence for each $M\in {{\mathbb{N}}}^{\ast }$. It is important to remark that all these coding schemes are lossless in the sense of definition 1.

As it is well known in classical data compression, the Kraft-McMillan inequality gives a necessary and sufficient condition for the existence of a uniquely decodable code (see *e.g*.^2^,). This result has been originally extended to the quantum domain in^12^, introducing a particular formalism. We proceed here to obtain a *quantum version of the Kraft-McMillan inequality*, compatible with the previous construction.

Let us first introduce the *length observable*, which allows to get a further notion of codeword length.

#### **Definition 2.**

The length observable Λ acting on ${ {\mathcal F} }_{{\mathscr{A}}}$ is defined as

4 $${\rm{\Lambda }}\equiv \sum _{\ell \mathrm{=0}}^{\infty }\ell \,{{\rm{\Pi }}}_{\ell },$$

where ${{\rm{\Pi }}}_{\ell }$ denotes the orthogonal projector onto the subspace ${ {\mathcal H} }_{{\mathscr{A}}}^{\,\otimes \ell }\subset { {\mathcal F} }_{{\mathscr{A}}}$.

Now, the quantum Kraft-McMillan inequality reads as follows.

#### **Theorem 1.**

For any losless quantum encoding scheme *U* given by Eq. (1), the following inequality must be satisfied:

5 $${\rm{Tr}}({U}^{\dagger }{k}^{-{\rm{\Lambda }}}U)\le 1.$$

The proof of this theorem, which mainly relies in its classical counterpart, is given in the section Methods, along with the proofs of the subsequent theorems.

### Source coding and von Neumann entropy bounds

As in the classical case, we are interested in quantum codes that minimize the amount of resources involved. However, in the quantum case arises an extra difficulty to quantify the number of resources since there is no a unique way of defining the notion of length of a quantum codeword. For a given encoding scheme *U*, the standard definition of *quantum codeword length* is the following.

#### **Definition 3.**

 The quantum codeword length of $|\omega \rangle \equiv U|s\rangle$ for some $|s\rangle \in { {\mathcal H} }_{{\mathscr{S}}}$ is given by the expectation value

6 $$\ell (|\omega \rangle )\equiv \langle \omega |{\rm{\Lambda }}|\omega \rangle =\sum _{i\mathrm{=1}}^{d}{|\langle {e}_{i}|s\rangle |}^{2}{\ell }_{i}\mathrm{.}$$

Thus, from this definition, the codewords may not have definite length in the sense that they are not eigenstates of the length operator in the general case. For that reason a quantum code given by the encoding scheme (1) is sometimes called *quantum indeterminate-length code* ^12^.

As we have noticed, one can introduce another important measure of the length of a quantum codeword. One used in the literature is the *base length* ^13^:

#### **Definition 4.**

 The base length of a quantum codeword $|\omega \rangle \equiv U|s\rangle$ is given by

7 $$l(|\omega \rangle )\,\equiv \,{\rm{\max }}\{\ell \in {\mathbb{N}}|\langle \omega |{{\rm{\Pi }}}_{\ell }|\omega \rangle \ne 0\}\,\,\,=\mathop{{\rm{\max }}}\limits_{\{i\in \mathrm{\{1,}\ldots ,d\}|{\langle e}_{i}|s\rangle \ne \mathrm{0\}}}\{{\ell }_{i}\mathrm{\}.}$$

Notice that the base length plays a key role as it determines the minimum size of the quantum register necessary to store a quantum codeword.

The base length of a quantum codeword is an integer whereas the quantum codeword length is not, in general. However, there is a relation between both lengths given by $\ell (|\omega \rangle )={\sum }_{\ell \mathrm{=0}}^{l(\omega )}\ell \langle \omega |{{\rm{\Pi }}}_{\ell }|\omega \rangle \le l(|\omega \rangle ){\sum }_{\ell }\langle \omega |{\Pi }_{\ell }|\omega \rangle$. Immediately, one has $\ell (|\omega \rangle )\le l(|\omega \rangle )$, with equality if and only if $|\omega \rangle =U|s\rangle$ is an eigenstate of Λ, *i.e*., if |*s*〉 is an eigenstate of *U*.

Henceforth, we consider that the state of the quantum source ${\mathscr{S}}$ is given by the density operator *ρ*, *i.e*., a positive semi-definite operator of trace one acting on ${{\mathbb{C}}}^{d}$. We will write the density operator using the decomposition on ensemble’s states, *i.e*., $\rho ={\sum }_{n\mathrm{=1}}^{N}{p}_{n}|{s}_{n}\rangle \langle {s}_{n}|$, or equivalently, considering the spectral decomposition, *i.e*., $\rho ={\sum }_{i\mathrm{=1}}^{d}{\rho }_{i}|{\rho }_{i}\rangle \langle {\rho }_{i}|$, where *ρ*_*i*_ is the eigenvalue corresponding to the eigenstate $|{{\rho }}_{i}\rangle$. In addition, we will denote as

8 $$C(\rho )\equiv U\rho {U}^{\dagger }=\sum _{i,i^{\prime} \mathrm{=1}}^{d}\langle {e}_{i}|\rho |{e}_{{i}^{^{\prime} }}\rangle |c(i)\rangle \langle c(i^{\prime} )|$$

the output of the quantum encoder (1). Then, according to definition 3, the *average codeword length* of ${\mathscr{S}}$ is given by

9 $$\ell (C(\rho ))\equiv {\rm{Tr}}(C(\rho ){\rm{\Lambda }})=\sum _{n\mathrm{=1}}^{N}{p}_{n}\sum _{i\mathrm{=1}}^{d}{|\langle {e}_{i}|{s}_{n}\rangle |}^{2}{\ell }_{i}\mathrm{.}$$

On the other hand, according to definition 4, the *base length* of ${\mathscr{S}}$ is

10 $$l(C(\rho ))\,\equiv \,{\rm{\max }}\,{\{l(U|{s}_{n}\rangle )\}}_{n\mathrm{=1}}^{N}=\,{\rm{\max }}\,{\{\mathop{{\rm{\max }}}\limits_{\{i\in \mathrm{\{1,}\ldots ,d\}|{\langle e}_{i}||{s}_{n}\rangle \ne \mathrm{0\}}}\{{\ell }_{i}\}\}}_{n\mathrm{=1}}^{N}\mathrm{.}$$

We have now all the ingredients to introduce *optimal quantum lossless codes*.

#### **Definition 5.**

 A quantum encoding scheme *U* is optimal for the source ${\mathscr{S}}$ if it minimizes the average codeword length, that is,

11 $${U}^{{\rm{opt}}}\equiv \mathop{{\rm{argmin}}}\limits_{{\rm{Tr}}({U}^{\dagger }{k}^{-{\rm{\Lambda }}}U)\le 1}{\rm{Tr}}(U\rho {U}^{\dagger }{\rm{\Lambda }})$$

and thus the minimal average codeword length for the source ${\mathscr{S}}$ is given by

12 $$\ell ({C}^{{\rm{opt}}}(\rho ))={\rm{Tr}}({C}^{{\rm{opt}}}(\rho ){\rm{\Lambda }}),$$

where $${{\rm{C}}}^{{\rm{opt}}}(\rho )\equiv {U}^{{\rm{opt}}}\rho {U}^{{\rm{opt}}\dagger }$$.

In the classical setting to search for the optimal code, one has to find for the set of integers $\{{\ell }_{i}\}$ that minimizes the averaged length subjected to the Kraft-McMillan inequality. It is well known that *Huffman code* provides the optimal solution^21^. Let us see that the quantum optimal code or the quantum version of Huffman code is obtained for an encoding scheme *U* with basis given by the eigenstates of *ρ* and the classical code *c* given by the Huffman code for the symbols $\mathrm{\{1,}\ldots ,d\}$ with probabilities given by the eigenvalues of $\rho$.

#### **Theorem 2.**

*The optimal quantum code of the quantum source* ${\mathscr{S}}$ *writes*

13 $${U}^{{\rm{opt}}}=\sum _{i\mathrm{=1}}^{d}|{c}^{{\rm{opt}}}(i)\rangle \langle {\rho }_{i}|,$$

*where* $\{{c}^{{\rm{opt}}}(i)\}$ *is the classical optimal code given by the Huffman code* ^21^ *of the symbols* $\mathrm{\{1,}\ldots ,d\}$ *with corresponding probabilities* $\{{\rho }_{1},\ldots ,{\rho }_{d}\}$.

Let us recall that there is no an analytic formula for the individual lengths ${\ell }_{i}$ of the classical Huffman code in the general case. On the other hand, if one drops the integer restriction of $\{{\ell }_{i}\}$ in the minimization problem, one obtains the optimum “lengths” $-{\mathrm{log}}_{k}{\rho }_{i}$. To take integer values, one can consider the excess integer part of these values, ${\ell }_{i}=\lceil -{\mathrm{log}}_{k}{\rho }_{i}\rceil$, and construct a corresponding code using the Kraft tree (see^2^ for more details). This is a well known method called the *Shannon coding* for which the average length is close to the optimal one (which is given by the Huffman code). Accordingly, we can say that the quantum version of the Shannon code is given by an encoding scheme of the form (13), where the classical code *c* is now given by the Shannon code. Nevertheless, without explicitly expressing the optimal code, it is possible to upper and lower bound the optimal average codeword length in terms of the von Neumann entropy of the source, as previously proved in a different formalism in^12^.

#### **Theorem 3.**

*The average length of the optimal code is lower and upper bounded as follows*

14 $$S(\rho )\le \ell ({C}^{{\rm{opt}}}(\rho )) < S(\rho )+\mathrm{1,}$$

*where* $S(\rho )=-{\rm{Tr}}(\rho {\mathrm{log}}_{k}\rho )$ *is the von Neumann entropy of a density operator ρ, and* ${\mathrm{log}}_{k}$ *is the logarithm of base k*.

According to theorem 3, the entropy of the source bounds the compression capacity. Moreover, one can attain the lower bound for the case of *K* independent and identical preparations of the source for large *K*. Let ${\rho }^{\otimes K}$ be the corresponding density operator, and denote by $\frac{1}{K}\,\ell ({C}^{{\rm{opt}}}({\rho }^{\otimes K}))$ the optimal average length code per source, where $C({\rho }^{\otimes K})$ is defined via the concatenation (2). Then, from $S({\rho }^{\otimes K})=KS(\rho )$ and theorem 3 one has $S(\rho )\le \frac{1}{K}\ell ({C}^{{\rm{opt}}}({\rho }^{\otimes K})) < S(\rho )+\frac{1}{K}$, so that

15 $$\mathop{\mathrm{lim}}\limits_{K\to \infty }\,\frac{1}{K}\ell ({C}^{{\rm{opt}}}({\rho }^{\otimes K}))=S(\rho \mathrm{).}$$

We end this section discussing what happens to the average codeword length when the encoding scheme is designed for a “wrong” density operator *τ* instead of the correct one $\rho$. This could be useful for the case where *τ* is the best estimation of the state of the source for instance. In such a situation, the average code length of the quantum Shannon code corresponding to *τ* is again bounded, as follows (see, *e.g*., refs^19,20^).

#### Theorem **4.**

*Let τ be a density operator whose diagonal form is* $\tau ={\sum }_{i\mathrm{=1}}^{d}{\tau }_{i}|{\tau }_{i}\rangle \langle {\tau }_{i}|$ *. Let us consider the quantum Shannon code* ${U}^{{\rm{Sh}}}={\sum }_{i\mathrm{=1}}^{d}|c(i)\rangle \langle {\tau }_{i}|$ *designed for τ, where* $c(i)$ *are classical codewords of the Shannon code, with lengths* ${\ell }_{i}=\lceil -{\mathrm{log}}_{k}{\tau }_{i}\rceil$ *. The average length of such a quantum encoding is bounded as follows*

16 $$S(\rho )+S(\rho \parallel \tau )\le \ell ({C}^{{\rm{Sh}}}(\rho )) < S(\rho )+S(\rho \parallel \tau )+\mathrm{1,}$$

*where* $${C}^{{\rm{Sh}}}(\rho )\equiv {U}^{{\rm{Sh}}}\rho {U}^{{{\rm{Sh}}}^{\dagger }}$$.

Notice that this gives an operational interpretation to the quantum relative entropy as follows: $S(\rho \Vert \tau )$ measures the deviation from the average codeword length of the quantum Shannon code, when the code is designed using a density operator which differs from density operator associated to the source (see also^22,23^, for a further understanding of the role of quantum relative entropy in the context of data compression).

### Source coding and quantum Rényi entropy bounds

Let us first note that the definition 5 of optimal code and the results given above are closely linked to the standard definition 3 of the length of a quantum codeword. However, there could be problems for which the relevant measure of length is not the usual one. In this sense, Müller *et al*. have used the average of the base lengths of the source in order to define a different optimal code^18^ and have obtained a complementary result to the one given by theorem 3. In this section we follow an alternative strategy, which is based on an extension of Campbell’s proposal to the quantum case^5^. Let us first introduce a notion of *exponential* quantum codeword length. The standard quantum codeword and base lengths turn out to be particular cases of our definition.

#### Definition **6.**

 The t-exponential length of a quantum codeword $|\omega \rangle \equiv U|s\rangle$ for some $|s\rangle \in { {\mathcal H} }_{{\mathscr{S}}}$ is given by the expectation value

17 $${\ell }_{t}(|\omega \rangle )\equiv \frac{1}{t}{\mathrm{log}}_{k}\langle \omega |{k}^{t{\rm{\Lambda }}}|\omega \rangle =\frac{1}{t}{\mathrm{log}}_{k}(\sum _{i\mathrm{=1}}^{d}{\langle {e}_{i}|s\rangle }^{2}{k}^{t{\ell }_{i}}),$$

where $t\ge 0$ is a parameter related to the cost assigned to large codewords. In the limiting cases, one has

18 $${\ell }_{0}(|\omega \rangle )\equiv \mathop{\mathrm{lim}}\limits_{t\to 0}{\ell }_{t}(|\omega \rangle )=\ell (|\omega \rangle )\quad \quad \,{\rm{and}}\,\quad \quad {\ell }_{\infty }(|\omega \rangle )\equiv \mathop{\mathrm{lim}}\limits_{t\to \infty }{\ell }_{t}(|\omega \rangle )=l(|\omega \rangle \mathrm{).}$$

Notice that $t\mapsto {\ell }_{t}(\omega )$ is a continuous nondecreasing function, *i.e*., ${\ell }_{t}(|\omega \rangle )\le {\ell }_{{t}^{^{\prime} }}(|\omega \rangle )$ for $t\le t^{\prime}$. Thus, by changing the parameter *t*, one can move continuously and increasingly from the standard quantum codeword length to the base length. In other words, the *t*-exponential codeword length will allow to make a compromise between minimizing the average length and the base length. Finally, note that if $|\omega \rangle \in {{\mathbb{C}}}^{\ell }$, *i.e*., the quantum codeword is an eigenstate of the length observable, then ${\ell }_{t}(|\omega \rangle )=\ell$, which is a reasonable property for a quantum codeword length measure.

According to definition 6, the *t*-exponential average codeword length of the quantum source ${\mathscr{S}}$ is given by

19 $${\ell }_{t}(C(\rho ))\equiv \frac{1}{t}{\mathrm{log}}_{k}{\rm{Tr}}(C(\rho )\,{k}^{t{\rm{\Lambda }}})=\frac{1}{t}{\mathrm{log}}_{k}(\sum _{n\mathrm{=1}}^{N}{p}_{n}\sum _{i\mathrm{=1}}^{d}\,{|\langle {e}_{i}|{s}_{n}\rangle |}^{2}{k}^{t{\ell }_{i}})\mathrm{.}$$

We introduce now the notion of optimal quantum code corresponding to our previously defined *t*-exponential codeword length. A natural choice is as follows:

#### **Definition 7.**

 A quantum encoding scheme *U* is *t*-exponential optimal for the source ${\mathscr{S}}$ if it minimizes the *t*-exponential average codeword length, that is,

20 $${U}_{t}^{{\rm{opt}}}\equiv \mathop{{\rm{argmin}}}\limits_{{\rm{Tr}}({U}^{\dagger }{k}^{-{\rm{\Lambda }}}U)\le 1}\,\frac{1}{t}{\mathrm{log}}_{k}{\rm{Tr}}(U\rho {U}^{\dagger }{k}^{t{\rm{\Lambda }}})$$

and thus the minimal *t*-exponential average codeword length for the source ${\mathscr{S}}$ is given by

21 $${\ell }_{t}({C}_{t}^{{\rm{opt}}}(\rho ))\,=\frac{1}{t}{\mathrm{log}}_{k}{\rm{Tr}}({C}_{t}^{{\rm{opt}}}(\rho ){k}^{t{\rm{\Lambda }}}),$$

where $${C}_{t}^{{\rm{opt}}}(\rho )\equiv {U}_{t}^{{\rm{opt}}}\rho {U}_{t}^{{\rm{opt}}\dagger }$$.

In the classical setting to search for the *t*-exponential optimal code, as for the standard context, one has to look for the set of integers $\{{\ell }_{i}\}$ that minimizes the *t*-exponential averaged length subjected to the Kraft-McMillan inequality. This problem has been already solved in^7,24,25^. In the quantum context, we prove here that the optimal code is again obtained for an encoding scheme *U* with basis given by the eigenstates of *ρ* and the classical *t*-exponential optimal code ${c}_{t}$ for the symbols $\mathrm{\{1,}\ldots ,d\}$ with probabilities given by the eigenvalues of *ρ*.

#### **Theorem 5.**

*The quantum code that minimizes the t-exponential average codeword length of the quantum source* ${\mathscr{S}}$ *writes*

22 $${U}_{t}^{{\rm{opt}}}=\sum _{i\mathrm{=1}}^{d}|{c}_{t}^{{\rm{opt}}}(i)\rangle \langle {\rho }_{i}|,$$

*where* $\{{c}_{t}^{{\rm{opt}}}(i)\}$ *is the classical code minimizing the t-exponential average code length of the symbols* $\mathrm{\{1,}\ldots ,d\}$ *with corresponding probabilities* $\{{\rho }_{1},\ldots ,{\rho }_{d}\}$.

As for the standard case, there is no an analytic formula for the individual optimal integer lengths ${\ell }_{i}$ leading to the minimum *t*-exponential average length of the classical code. But, again, if one drops the integer restriction of $\{{\ell }_{i}\}$ in the minimization problem, one obtains now the optimum “lengths” $-{\mathrm{log}}_{k}\,{\rho }_{{t}_{i}}$ where the ${\rho }_{{t}_{i}}$ are the “escort probabilities”, eigenvalues of the “escort” density operator

23 $${\rho }_{t}\equiv \frac{{\rho }^{\frac{1}{1+t}}}{{\rm{Tr}}{\rho }^{\frac{1}{1+t}}},$$

acting on ${ {\mathcal H} }_{{\mathscr{S}}}$. To take integer values, one can again consider the excess integer part of these values, ${\ell }_{i}=\lceil -{\mathrm{log}}_{k}{\rho }_{{t}_{i}}\rceil$, and construct a corresponding code using the Kraft tree, that is the Shannon code corresponding to the escort probabilities $\{{\rho }_{{t}_{i}}\}$. However, independently of the explicit expression of the generalized optimal code (20), it is possible to upper and lower bound the optimal *t*-exponential average quantum codeword length (21) in terms of the quantum Rényi entropy of the source.

#### Theorem **6.**

*The t-exponential average length of the t-exponential optimal code is lower and upper bounded as follows*

24 $${S}_{\frac{1}{1+t}}(\rho )\le {\ell }_{t}({C}_{t}^{{\rm{opt}}}(\rho )) < {S}_{\frac{1}{1+t}}(\rho )+\mathrm{1,}$$

*where* ${S}_{\alpha }(\rho )=\frac{1}{1-\alpha }{\mathrm{log}}_{k}{\rm{Tr}}{\rho }^{\alpha },\,\alpha \ge 0$, *is the quantum Rényi entropy of the density operator of the source* $\rho$.

We recall that our aim is to provide a scheme to address the problem of how to codify codewords of a quantum source allowing chains of variable length, but considering a penalization for large codewords. This aim can be achieved by appealing to definitions 6 and 7 and theorems 5 and 6. In particular, we can interpret theorem 6 as the quantum version of Campbell’s source coding theorem^5^. Hence, the quantum Rényi entropy plays a role similar to that of von Neumann’s in the standard quantum source coding theorem, when an exponential penalization is considered. Indeed, theorem 3 results as a particular case of our theorem 6 (with $t=0$), recovering the results of Schumacher and Westmoreland^12^. This situation is completely analogous to that of the classical setting, with regard to the roles played by Rényi and Shannon measures for the cases with and without penalization, respectively. Consequently, this allows us to provide a natural operational interpretation for the quantum Rényi entropy in relation with the problem of lossless quantum data compression. Finally, notice that this is an alternative approach to that of Müeller *et al*.^18^, where they have studied an analogous problem, but minimizing the average of the individual base lengths of the source instead of considering a penalization over large codewords.

According to theorem 6, the quantum Rényi entropy of the source bounds the compression capacity when an exponential penalization is considered. As in the case with no penalization, one can attain the lower bound for the case of *K* independent and identically prepared sources for large *K*. Thus, consider a density operator ${\rho }^{\otimes K}$ and denote by $\frac{1}{K}\,{\ell }_{t}({C}_{t}^{{\rm{opt}}}({\rho }^{\otimes K}))$ to the $t$-exponential optimal average length code per source. Then, using that ${S}_{\alpha }({\rho }^{\otimes K})=K{S}_{\alpha }(\rho )$ and theorem 6, one has ${S}_{\frac{1}{1+t}}(\rho )\le \frac{1}{K}{\ell }_{t}({C}_{t}^{{\rm{opt}}}({\rho }^{\otimes K})) < {S}_{\frac{1}{1+t}}(\rho )+\frac{1}{K}$. In this way

25 $$\mathop{\mathrm{lim}}\limits_{K\to \infty }\,\frac{1}{K}{\ell }_{t}({C}_{t}^{{\rm{opt}}}({\rho }^{\otimes K}))\,={S}_{\frac{1}{1+t}}(\rho \mathrm{).}$$

Let us point out that the quantum Rényi entropy appears also naturally in the determination of the exponent of the average error of the quantum fixed-length source coding^26,27^, which is closely related to the Chernoff exponent appearing in classical discrimination problems. This exponent provides thus another interpretation of the quantum Rényi entropy. Our approach differs in that we study the role played by the quantum Rényi entropy in the problem of lossless quantum data compression with penalization.

As in the end of the previous section, we now discuss what happens with the *t*-exponential average codeword length when the encoding scheme is designed for a density operator $\tau$, *i.e*., using the escort density operator ${\tau }_{t}\equiv \frac{{\tau }^{\frac{1}{1+t}}}{{\rm{Tr}}\,{\tau }^{\frac{1}{1+t}}}$. In that case the *t*-exponential average codeword length of the quantum Shannon code corresponding to ${\tau }_{t}$ is again bounded as follows.

#### Theorem **7.**

*Let* $\tau$ *be a density operator whose diagonal form is* $\tau ={\sum }_{i\mathrm{=1}}^{d}{\tau }_{i}|{\tau }_{i}\rangle \langle {\tau }_{i}|$ *. Let us consider the quantum Shannon code* ${U}_{t}^{{\rm{Sh}}}={\sum }_{i\mathrm{=1}}^{d}|c(i)\rangle \langle {\tau }_{i}|$ *designed for the escort density operator* ${\tau }_{t}$ *, where* $c(i)$ *are classical codewords of the Shannon code, with lengths* ${\ell }_{i}=\lceil -{\mathrm{log}}_{k}{\tau }_{{t}_{i}}\rceil$ *. The* $t$ *-exponential average length of such a quantum encoding is bounded as follows*

26 $${S}_{\frac{1}{1+t}}(\rho )+{S}_{1+t}({\rho }_{t}\parallel {\tau }_{t})\le {\ell }_{t}({C}_{t}^{{\rm{Sh}}}(\rho )) < {S}_{\frac{1}{1+t}}(\rho )+{S}_{1+t}({\rho }_{t}\parallel {\tau }_{t})+\mathrm{1,}$$

*where* $${C}_{t}^{{\rm{Sh}}}(\rho )\equiv {U}_{t}^{{\rm{Sh}}}\rho {U}_{t}^{{\rm{Sh}}\dagger }$$.

It is important to remark that this theorem provides an operational interpretation for the quantum Rényi divergence as follows: ${S}_{1+t}({\rho }_{t}\parallel {\tau }_{t})$ quantifies the deviation from the *t*-exponential average codeword length of the quantum Shannon code, when the code is designed using an escort density operator which differs from density operator associated to the source.

It would be desirable to have some expression that indicates how the standard average and the base length of the *t*-exponential optimal code behave when an exponential penalization is considered. However, this is not possible as there is no an analytic formula for the individual codeword length in this case, in general. An interesting alternative is analyzing how the the standard average of the quantum Shannon code is affected by an exponential penalization.

#### **Theorem 8.**

 Let ${U}_{t}^{{\rm{Sh}}}={\sum }_{i\mathrm{=1}}^{d}|c(i)\rangle \langle {\rho }_{i}|$ be the quantum Shannon code designed for the escort density operator ${\rho }_{t}$, for which the classical codewords lengths are given by ${\ell }_{i}=\lceil -{\mathrm{log}}_{k}{\rho }_{{t}_{i}}\rceil$. The average length of this code is bounded as follows

27 $$\frac{1}{1+t}S(\rho )+\frac{t}{1+t}{S}_{\frac{1}{1+t}}(\rho )\,\le \,\ell ({C}_{t}^{{\rm{Sh}}}(\rho )) < \frac{1}{1+t}S(\rho )+\frac{t}{1+t}{S}_{\frac{1}{1+t}}(\rho )+1.$$

Notice that the bounds are basically a convex combination of the von Neumann entropy (related to the minimum average length) and the Rényi entropy (related to the minimal $t$-exponential average length) of the source. Since ${S}_{\frac{1}{1+t}}(\rho )\ge S(\rho )$, the average length $\ell ({C}_{t}^{{\rm{Sh}}}(\rho ))$ increases with respect to *t*; in particular, $\ell ({C}_{t}^{{\rm{Sh}}}(\rho ))\ge \ell ({C}^{{\rm{Sh}}}(\rho ))$. On the contrary, for the base length, one can see that when ${\rho }_{i}$ is small enough, there exists a parameter *t* sufficiently large so that $l({C}_{t}^{{\rm{Sh}}}(\rho )) < l({C}^{{\rm{Sh}}}(\rho ))$. In particular, the base length can be lessen up to $\lceil {S}_{0}(\rho )\rceil =\lceil {\mathrm{log}}_{k}{\rm{rank}}\rho \rceil$. So, there is a tradeoff between $\ell ({C}_{t}^{{\rm{Sh}}}(\rho ))$ and $l({C}_{t}^{{\rm{Sh}}}(\rho ))$ with respect to *t*. The optimal choosing of the cost parameter depends on the particularities of the problem in question (e.g., the size of the quantum register, etc). Finally, notice that for the exceptional case that all $-{\mathrm{log}}_{k}{\rho }_{ti}$ are integers, the quantum Shannon code hence designed coincides with the *t*-exponential optimal code ${C}_{t}^{{\rm{opt}}}$ of theorem 5 and the lower bound of (24) is achieved.

## Discussion

We have addressed the problem of lossless quantum data compression. In particular, we have considered the case in which codification of large codewords is penalized. Our work can be regarded as a quantum version of Campbell’s work^5^.

First, we have provided an expression for the optimal code for the case with exponential penalization (theorem 5) in terms of its classical counterpart^7,24,25^. We have shown that this penalization affects the optimal code in such a way that the Rényi entropy of the source bounds the *t*-exponential average codeword length (theorem 6). As a corollary, in the limit of a large number of independent and identically prepared sources, we have found that the capacity of compression equals the Rényi entropy of the source. Thus, the quantum Rényi entropy acquires a natural operational interpretation. In addition, we have found that a wrong description of the source produces an excess term in the bound of the average codeword length, which is related to the quantum Rényi divergence (theorem 7). Given that we recover the results by Schumacher and Westmoreland^12^ when penalization is negligible, our work can be seen as an generalization of theirs.

Finally, we have discussed how the average and base lengths of the quantum Shannon code behave in terms of the cost parameter, which is related to the penalization (theorem 8). Indeed, there is a tradeoff between these two quantities, in the sense that it is possible to reduce the base length, but with the side effect of increasing the average length and viceversa.

It is worth noticing that our approach provides an alternative to that of Müeller *et al*.^18^, where they have studied an analogous problem, but minimizing the average of the individual base lengths of the source. Our results are complementary to theirs.

## Methods

In this section, we give the proofs of all theorems.

Proof of theorem 1.

### *Proof*

 Notice first that ${U}^{\dagger }{k}^{-{\rm{\Lambda }}}U={\sum }_{i\mathrm{=1}}^{d}{k}^{-{\ell }_{i}}|{e}_{i}\rangle \langle {e}_{i}|$ due to

28 $$\langle c(i)|{{\rm{\Pi }}}_{\ell }|c(i^{\prime} )\rangle ={\delta }_{\ell ,{\ell }_{i}^{\prime} }\,{\delta }_{{{i,i}}^{^{\prime} }},$$

where the ${\ell }_{i}$ are the lengths of the classical codewords $c(i)$. Then, one directly obtains ${\rm{Tr}}({U}^{\dagger }{k}^{-{\rm{\Lambda }}}U)={\sum }_{i\mathrm{=1}}^{d}{k}^{-{\ell }_{i}}$. Given that the code *c* is uniquely decodable, the proof ends by appealing to the classical Kraft-McMillan inequality.

Proof of theorem 2.

### *Proof*

 Let us first notice that the quantum Kraft-McMillan constraint is independent of the basis $\{|{e}_{i}\rangle \}$ of a given lossless quantum encoding scheme $U={\sum }_{i}|c(i)\rangle \langle {e}_{i}|$. Then, to prove the theorem one can do it in two steps. On the one hand, let us first fix a classical code $c$ and minimize $\ell (C(\rho ))={\sum }_{i,j}{\ell }_{i}{\rho }_{j}{\langle {e}_{i}|{\rho }_{j}\rangle }^{2}$ over the set of basis of ${{\mathbb{C}}}^{d}$. Let us introduce the doubly stochastic matrix *D* with entries ${D}_{i,j}\equiv {|\langle {e}_{i}|{\rho }_{j}\rangle |}^{2}$, *i.e*., ${D}_{i,j}\ge 0$ and ${\sum }_{j}{D}_{i,j}={\sum }_{i}{D}_{i,j}=1$ for all *i* and *j*. So the minimization problem consists in minimizing $\overrightarrow{\ell }\,D\,{\overrightarrow{\rho }}^{t}$ over the set of doubly stochastic matrices, where $\overrightarrow{\ell }=[{\ell }_{1}\ldots {\ell }_{d}]$ and $\overrightarrow{\rho }=[{\rho }_{1}\ldots {\rho }_{d}]$. From the Birkhoff theorem^28,29^, one can write $D={\sum }_{k}{\pi }_{k}{{\rm{\Pi }}}_{k}$ as a convex combination of permutations matrices ${{\rm{\Pi }}}_{k}$. Thus, $\overrightarrow{\ell }\,D\,{\overrightarrow{\rho }}^{t}={\sum }_{k}{\pi }_{k}\overrightarrow{\ell }{{\rm{\Pi }}}_{k}{\overrightarrow{\rho }}^{t}\ge \overrightarrow{\ell }\,{{\rm{\Pi }}}_{{{\rm{k}}}^{^{\prime} }}{\overrightarrow{\rho }}^{t}$ for some $k^{\prime}$, so that $D={{\rm{\Pi }}}_{{{\rm{k}}}^{^{\prime} }}$. Although one does not know such permutation, this implies that each element of $\{|{e}_{i}\rangle \}$ coincides with only one of $\{|{\rho }_{j}\rangle \}$. On the other hand, one can skip the search of ${{\rm{\Pi }}}_{{{\rm{k}}}^{^{\prime} }}$ since one has now to minimize the averaged length with respect to the set of lengths $\{{\ell }_{i}\}$ subject to the classical Kraft-McMillan inequality. Indeed, without loss of generality, the permutation can be incorporated in the lengths by replacing $\overrightarrow{\ell ^{\prime} }\to \overrightarrow{\ell }\,{{\rm{\Pi }}}_{{{\rm{k}}}^{^{\prime} }}$. Therefore, one has $|{e}_{i}\rangle =|{\rho }_{i}\rangle$ and thus $\ell (C(\rho ))={\sum }_{i}{\ell }_{i}{\rho }_{i}$ is the classical average length of the classical code $c$. Finally, one has to find the classical optimal code $c$, whose solution is well known in the literature given by the Huffman code^21^.□

Proof of theorem 3.

### *Proof*

 Let us first introduce the density operator

29 $$\sigma \,\equiv \,\frac{{U}^{\dagger }{k}^{-{\rm{\Lambda }}}U}{\beta }\quad \quad \,{\rm{with}}\,\quad \quad \beta \,\equiv {\rm{Tr}}({U}^{\dagger }{k}^{-{\rm{\Lambda }}}U)$$

acting on ${ {\mathcal H} }_{{\mathscr{S}}}$. Let $C(\rho )=U\rho {U}^{\dagger }$ with *U* an arbitrary encoding scheme of the form (1). Then, noting that ${\rm{\Lambda }}=-{\mathrm{log}}_{k}{k}^{-{\rm{\Lambda }}}$, and thus that ${U}^{\dagger }{\rm{\Lambda }}U=-{\mathrm{log}}_{k}({U}^{\dagger }{k}^{-{\rm{\Lambda }}}U)$, it is straightforward to show that

30 $$\ell (C(\rho ))=S(\rho )+S(\rho \parallel \sigma )-{\mathrm{log}}_{k}\beta ,$$

where $S(\rho \parallel \sigma )={\rm{Tr}}[\rho ({\mathrm{log}}_{k}\rho -{\mathrm{log}}_{k}\sigma )]$ is the quantum relative entropy. The quantum relative entropy being definite positive, and from ${\mathrm{log}}_{k}\beta \le 0$ due to the quantum Kraft-McMillan inequality, it follows that

31 $$\ell (C(\rho ))\ge S(\rho ),$$

for any encoding scheme *U*, in particular for the optimum one.

In order to proof the upper bound, let us consider the quantum Shannon code ${U}^{{\rm{Sh}}}={\sum }_{i\mathrm{=1}}^{d}|c(i)\rangle \langle {\rho }_{i}|$ of $\rho$, where the lengths of the codewords $\{c(i)\}$ are $\{{\ell }_{i}=\lceil -{\mathrm{log}}_{k}{\rho }_{i}\rceil \}$. Notice that this code satisfies the quantum Kraft-McMillan inequality (5) by construction. Then, from $\lceil -{\mathrm{log}}_{k}{\rho }_{i}\rceil  < -{\mathrm{log}}_{k}{\rho }_{i}+1$, we have

32 $$\ell ({C}^{{\rm{Sh}}}(\rho ))=\sum _{i\mathrm{=1}}^{d}{\rho }_{i}\lceil -{\mathrm{log}}_{k}{\rho }_{i}\rceil  < S(\rho )+1.$$

The upper bound in (14) immediately follows from this inequality and from $\ell ({C}^{{\rm{opt}}}(\rho ))\le \ell ({C}^{{\rm{Sh}}}(\rho ))$ (by definition of the optimal code).□

Proof of theorem 4.

### *Proof*

Let ${U}^{{\rm{Sh}}}={\sum }_{i\mathrm{=1}}^{d}|c(i)\rangle \langle {\tau }_{i}|$ be the quantum Shannon code of *τ*. It is straightforward to show that $\ell ({C}^{{\rm{Sh}}}(\rho ))={\sum }_{i\mathrm{=1}}^{d}\langle {\tau }_{i}|\rho |{\tau }_{i}\rangle \lceil -{\mathrm{log}}_{k}{\tau }_{i}\rceil$. The bounds result thus directly from $-{\mathrm{log}}_{k}{\tau }_{i}\le \lceil -{\mathrm{log}}_{k}{\tau }_{i}\rceil  < -{\mathrm{log}}_{k}{\tau }_{i}+1$ and $-{\sum }_{i\mathrm{=1}}^{d}\langle {\tau }_{i}|\rho |{\tau }_{i}\rangle {\mathrm{log}}_{k}{\tau }_{i}=-{\rm{Tr}}(\rho {\mathrm{log}}_{k}\tau )=S(\rho )+S(\rho \parallel \tau )$.□

Proof of theorem 5.

### *Proof*

For a given lossless quantum encoding scheme $U={\sum }_{i}|c(i)\rangle$〈${e}_{i}|$ it is straightforward to see that ${\ell }_{t}(C(\rho ))=\frac{1}{t}{\mathrm{log}}_{k}({\sum }_{i,j}{k}^{t{\ell }_{i}}{\rho }_{j}|\langle {e}_{i}|{\rho }_{j}\rangle {|}^{2})$. Noting that minimizing ${\ell }_{t}(C(\rho ))$ is equivalent to minimizing ${\sum }_{i,j}{k}^{t{\ell }_{i}}{\rho }_{j}|\langle {e}_{i}|{\rho }_{j}\rangle {|}^{2}$, the proof is the very same than that of theorem 2, where $\overrightarrow{\ell }$ is replaced by $[{k}^{t{\ell }_{1}}\ldots {k}^{t{\ell }_{d}}]$ and where the classical optimal code turns to be $\{{c}_{t}^{{\rm{opt}}}(i)\}$. This last one can be computed by the algorithms proposed in^7,24,25^. □

Proof of theorem 6.

### *Proof*

The proof is similar to that of theorem 3. Let $C(\rho )=U\rho {U}^{\dagger }$ with *U* an arbitrary encoding scheme of the form (1). Then, noting that $\rho {U}^{\dagger }{k}^{t{\rm{\Lambda }}}U=\rho {({U}^{\dagger }{k}^{-{\rm{\Lambda }}}U)}^{-t}={\rho }_{t}^{1+t}{\sigma }^{-t}{\beta }^{-t}{[{\rm{Tr}}({\rho }^{\frac{1}{1+t}})]}^{\mathrm{(1}+t)}$, where $\sigma$ and $\beta$ are defined in (29) and ${\rho }_{t}$ in (23), we immediately obtain

33 $${\ell }_{t}(C(\rho ))={S}_{\frac{1}{1+t}}(\rho )+{S}_{1+t}({\rho }_{t}\parallel \sigma )-{\mathrm{log}}_{k}\beta ,$$

where ${S}_{\alpha }(\rho \parallel \sigma )=\frac{1}{\alpha -1}{\mathrm{log}}_{k}{\rm{Tr}}{\rho }^{\alpha }{\sigma }^{1-\alpha }$ is the quantum Rényi divergence (see *e.g*.^30^). The quantum Rényi divergence being definite positive, and from ${\mathrm{log}}_{k}\beta \le 0$ due to the quantum Kraft-McMillan inequality, it follows that

34 $${\ell }_{t}(C(\rho ))\ge {S}_{\frac{1}{1+t}}(\rho ),$$

for any encoding scheme *U*, in particular for the optimal one.

In order to prove the upper bound, let us now consider the quantum Shannon code ${U}_{t}^{{\rm{Sh}}}={\sum }_{i\mathrm{=1}}^{d}|c(i)\rangle \langle {\rho }_{i}|$ of the escort density operator ${\rho }_{t}$, where the lengths of the codewords $\{c(i)\}$ are $\{{\ell }_{i}=\lceil -{\mathrm{log}}_{k}{\rho }_{{t}_{i}}\rceil \}$ being ${\rho }_{{t}_{i}}$ the escort probabilities, eigenvalues of ${\rho }_{t}$. Notice that this code satisfies the quantum Kraft-McMillan inequality (5) by construction. Then, from $\lceil -{\mathrm{log}}_{k}{\rho }_{{t}_{i}}\rceil  < -{\mathrm{log}}_{k}{\rho }_{{t}_{i}}+1$, we have

35 $${\ell }_{t}({C}_{t}^{{\rm{Sh}}}(\rho ))=\frac{1}{t}{\mathrm{log}}_{k}(\sum _{i\mathrm{=1}}^{d}{\rho }_{i}{k}^{t\lceil -{\mathrm{log}}_{k}{\rho }_{{t}_{i}}\rceil })\, < \,{S}_{\frac{1}{1+t}}(\rho )+1.$$

Because ${\ell }_{t}({C}_{t}^{{\rm{opt}}}(\rho ))\le {\ell }_{t}({C}_{t}^{{\rm{Sh}}}(\rho ))$ by definition of the optimal code, the upper bound in (24) immediately follows from this inequality.□

Proof of theorem 7.

### *Proof*

Let ${U}_{t}^{{\rm{Sh}}}={\sum }_{i\mathrm{=1}}^{d}|c(i)\rangle \langle {\tau }_{i}|$ be the quantum Shannon code of the escort density operator ${\tau }_{t}$. It is straightforward to show that ${\ell }_{t}({C}_{t}^{{\rm{Sh}}}(\rho ))=\frac{1}{t}{\mathrm{log}}_{k}({\sum }_{i\mathrm{=1}}^{d}\langle {\tau }_{i}|\rho |{\tau }_{i}\rangle {k}^{t\lceil -{\mathrm{log}}_{k}{\tau }_{{t}_{i}}\rceil })$. The bounds result thus directly from $-{\mathrm{log}}_{k}{\tau }_{{t}_{i}}\le \lceil -{\mathrm{log}}_{k}{\tau }_{{t}_{i}}\rceil \, < -{\mathrm{log}}_{k}{\tau }_{{t}_{i}}+1$ together with $\sum _{i\mathrm{=1}}^{d}\langle {\tau }_{i}|\rho |{\tau }_{i}\rangle {\tau }_{{t}_{i}}^{-t}={\rm{Tr}}(\rho {\tau }_{t}^{-t})={[{\rm{Tr}}({\rho }^{\frac{1}{1+t}})]}^{1+t}{\rm{Tr}}({\rho }_{t}^{1+t}{\tau }_{t}^{-t}).$    □

Proof of theorem 8

### *Proof*

Notice that for this code, ${U}_{t}^{{\rm{Sh}}}={\sum }_{i=1}^{d}|c(i)\rangle \langle {\rho }_{i}|$, the classical codewords lengths can be expressed as

36 $${\ell }_{i}=\lceil -{\mathrm{log}}_{k}{\rho }_{{t}_{i}}\rceil =\lceil \frac{1}{1+t}(-{\mathrm{log}}_{k}{\rho }_{i})+\frac{t}{1+t}{S}_{\frac{1}{1+t}}(\rho )\rceil \mathrm{.}$$

Thus, we have that the average length is given by

37 $$\ell ({C}_{t}^{{\rm{Sh}}}(\rho ))=\sum _{i\mathrm{=1}}^{d}{\rho }_{i}\lceil -{\mathrm{log}}_{k}{\rho }_{{t}_{i}}\rceil =\lceil \frac{1}{1+t}S(\rho )+\frac{t}{1+t}{S}_{\frac{1}{1+t}}(\rho )\rceil ,$$

so that the lower and upper bounds in (27) are directly obtained.
